# The Areas of Echinococcosis From the Qinghai-Tibet Plateau Extend to Yunnan: An Observation of Deworming and Control Integrated Impact Evaluation on Dogs

**DOI:** 10.1155/japr/4537456

**Published:** 2025-03-14

**Authors:** Ben-Fu Li, Dan Xiao, Xin-Liu Yan, Jin-Rong Zi, Jia Peng, Jian-Xiong Li, Xuan Cai, Qian Xu, Zheng-Qing Wang, Fang-Wei Wu, Ya-Ming Yang

**Affiliations:** ^1^Yunnan Institute of Parasitic Diseases, Yunnan Provincial Center of Malaria Research, Yunnan Provincial Collaborative Innovation Center for Public Health and Disease Prevention and Control, Yunnan Provincial Key Laboratory of Vector-borne Diseases Control and Research, Puer, China; ^2^Tibet Center for Disease Control and Prevention, Lhasa, China

**Keywords:** Echinococcosis, impact assessment, infectious source dog, the Qinghai-Tibet Plateau

## Abstract

**Background:** *Echinococcus granulosus* is a zoonotic parasitic disease caused by the larval stage of the parasite Echinococcus, which has a long incubation period, expensive diagnosis and treatment, and long duration of medication, so it brings a great economic burden to patients and their families. Shangri-La City is one of the counties with a high prevalence of echinococcosis in Yunnan Province. The antigen-positive rate of Echinococcus feces is high and there is a potential risk of infecting people. Evaluation of comprehensive prevention and control intervention and different frequencies of dewormed dogs (EC-DD) are crucial.

**Methods:** From 2022 to 2023, Jiefang, Nishi, and Jidi villages in Jiantang Town of Shangri-La City were selected as the intervention group (TIG) and Hongpo Village as the control group (TCG) for EC-DD. TIG took comprehensive intervention measures such as registration management of domestic dogs (RMDD), single tying of dogs alone (STDA), deworming of registered domestic dogs (DRDD), standardized disposal of feces after deworming (SDFD), and population health promotion and intervention (PHPI), while TCG did not do any prevention and control intervention.

**Results:** The evaluation survey results show that the positive rate of Echinococcus antigen (PREA) of TIG decreased from 3.15% baseline to 1.94%. Compared with the baseline, it decreased 38.41%, and the PREA of TCG increased from 1.88% baseline to 4.17%. Compared with the baseline, it increased 56.35%. The knowledge awareness rate (TKAR) was increased from 56.56% baseline to 81.46%. Compared with the baseline, it increased by 30.57%. In the survey of dog keeping, the evaluation values of STDA, DRDD, SDFD, and the practice of not feeding dogs the diseased organs of animals were higher than the baseline values. In the survey of people's behavior habits, the evaluation values of people regularly washing hands, not drinking raw water, and not burning cow dung at home also improved compared with the baseline value.

**Conclusions:** In areas with low prevalence of echinococcosis, the village with positive dogs shall take measures for the management of RMDD, STDA, and SDFD and perform deworming four times a year. In addition to these, the comprehensive prevention and control measures combined with PHPI can effectively control the PREA.

## 1. Introduction

The *Echinococcus granulosus* is a zoonotic parasitic disease caused by the larval stage of the parasite Echinococcus. It has been acknowledged as one of the world's most lethal parasitic zoonoses [1, 2]. People in epidemic areas commonly become poor or return to poverty because of echinococcosis, which is a major burden on communities in endemic areas of China and poses an important public health problem [3–9]. Human cystic echinococcosis (CE) and alveolar echinococcosis (AE) are caused by the larval stage of *E. granulosus* and *E. multilocularis*, respectively. The World Health Organization (WHO) listed echinococcosis, including both CE and AE, as a neglected tropical disease in 2010 [10].

China is a country affected heavily by human echinococcoses [11]. In China, the nationally estimated numbers of CE and AE cases explain 40% and 95% of the total global burden of the infections, respectively [12, 13]. Prevalence of AE was particularly in 9 provinces/autonomous regions (ARs) located in Western China: including Inner Mongolia Uighur AR, Sichuan, Tibet AR, Gansu, Qinghai, Ningxia Hui AR, Yunnan, Shaanxi, and Xinjiang [14–19]. Shangri-La City is one of the most severe epidemic of echinococcosis Counties/Cities in Yunnan Province. The positive rate of Echinococcus antigen (PREA) in dogs (10.65%) was higher than the national average (4.25%) [19, 20].

Dogs, wolves, foxes, etc. of canine animals are the primary end-hosts of echinococcus [11]. After ingesting diseased organs such as the liver and lungs of animals containing echinococcus. It develops into an adult worm in the small intestine, and the adult worm proglottids or eggs are excreted with the feces, thus polluting the natural environment such as pastures, water sources, and livestock products such as cattle and wool. People or livestock animals such as cattle, sheep, and pigs contact with infected dogs and live together in the polluted environment. Metacestode of echinococcus is infected by ingestion of eggs, which eventually leads to the spread of echinococcus [21–23].

In recent years, the prevalence of metacestode of echinococcus began to spread in the nonendemic areas of Yunnan Province due to the social economy, convenient communication and transportation, the increase of dogs raised by urban residents, the increase of stray dogs and their resale, and the trade of dogs, cattle, and livestock products in endemic areas [24]. Consequently, better surveillance and response tools are required to estimate and predict the real impact of these two diseases in Yunnan and to strengthen the implementation of prevention and control interventions in targeted high-risk areas [13]. In order to better control the spread of Echinococcus canis, we carried out comprehensive prevention and control measures such as RMDD, STDA, DRDD, and SDFD in the endemic area of Echinococcus canis in the Qinghai-Tibet Plateau to explore the best scheme for deworming dogs with echinococcus.

## 2. Methods

### 2.1. Study Site

Shangri-la is located in the northwest of Yunnan Province and the hinterland of the Hengduan Mountains on the southeast margin of the Qinghai-Tibet Plateau. It sits between 99°20⁣′ and 100°19⁣′ east longitude and 26°52⁣′ and 28°52⁣′ north latitude, in the confluence area of Yunnan, Sichuan, and Tibet, at an altitude of 3298 m. It borders on Daocheng County and Muli County of Sichuan Province to the east, Yulong County of Lijiang City, Yunnan Province to the southeast, Weixi County and Deqin County in Diqing Prefecture across the Jinsha River to the south, and Derong County and Xiangcheng County in Sichuan Province to the northwest. The total area of the city is 11,613 km^2^, encompassing 10 townships, 1 community, 62 administrative villages, and 719 villagers' groups. The permanent population of over 3 years old is 116,865 people, making it a semiagricultural and semipastoral area. By 2022, a total of 35 cases of echinococcosis had been reported in the city, with 9,495 domestic dogs serving as infection sources.

Among them, Jiantang Town is situated in the central-west of Shangri-La City, comprising a total of 5 communities and 5 administrative villages. The township residents have a per capita annual net income of 10,088 yuan. It is a pastoral area inhabited by 26 ethnic groups, including Tibetans, Naxi, Bai, Hui, and others. Jiefang, Jidi, and Nishi villages were selected as Target Intervention Villages (TIG), and Hongpo Village as a Target Control Village (TCG). There were 12,402 permanent residents and 1069 dogs in TIG, and 251 dogs in TCG.

**Figure 1:**
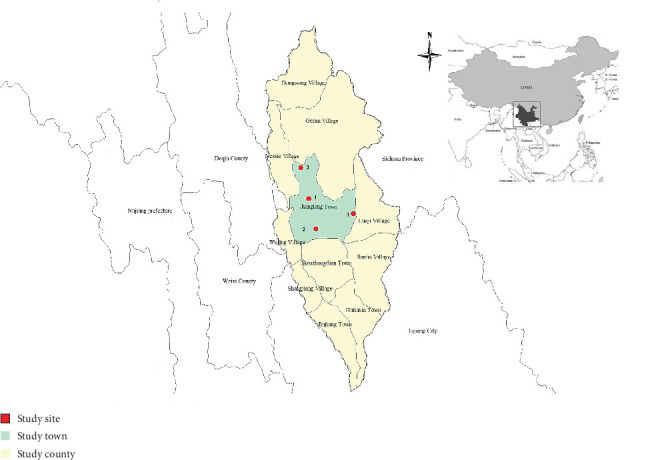
Map of the study site and neighboring region: (1) Jiefang Village, (2) Nishi Village, (3) Jidi Village, (4) Hongpo Village.

The area boasts a suitable ecological environment and climatic conditions, with numerous wild animals. This results in a complex transmission chain of echinococcosis and a large number of family dogs. As a production tool for Tibetan residents, domestic dogs are often reared in pasture with cattle, sheep, and Tibetan pigs, posing a risk of transmission between dogs and animals and the potential to infect humans (Figure 1).

### 2.2. Core Interventions

Based on the village as a unit, the number of domestic dogs was investigated, and TIG took measures such as RMDD, requiring dogs to be hitched and not released, prohibiting dogs from being kept in captivity with cows, sheep, and pigs, and ensuring dogs were hitched separately. According to the PREA results of the baseline investigation, villages with a PREA greater than 5% were dewormed monthly, for a total of 12 times a year. Villages with a PREA between 5% and 1% (inclusive) were dewormed once a quarter, for a total of 4 times. Villages with a PREA of less than 1% were dewormed twice a year, for a total of 2 times. Within 3 days of the SDFD, each intervention point conducted publicity through WeChat group sharing of echinococcosis prevention and control knowledge videos, on-site publicity, and distribution of publicity materials.

### 2.3. Study Procedures

According to the cases in Shangri-La, the TIG and TCG carry out a baseline investigation. The TIG implemented comprehensive prevention and control measures to promote intervention, while the TCG did not receive any intervention. One year later, the EC-DD included PREA, the knowledge awareness rate (TKAR) of human, as well as change in dog-rearing practics, life behavior, and attitude. Trained investigators used one-to-one question-and-answer format to conduct self-designed questionnaires. Local residents over 12 years old were randomly selected from each village for the survey. The number of residents surveyed in each village was not less than 100, and the total number of residents surveyed in four villages was not less than 400.

#### 2.3.1. Canine Infection

In each village, no fewer than 100 dog feces samples were randomly collected, and no less than 400 dog feces samples were collected in total from four villages. Dog feces samples were collected in plastic bags, with the dog owner's name, date, and place of collection being recorded. These samples were then sent to the laboratory in a cooler. The samples were inactivated in the refrigerator at −80°C for 7 days, Afterward, ELISA was used to detect the feces antigen of Echinococcus canis. The test kit was obtained from Tianzhitai Biotechnology Research Institute (Zhuhai) Co. Ltd. (Reagent batch numbers: 20202617 and 20220234), and the test procedure was carried out according to the instruction manual.

#### 2.3.2. Population Awareness Rate

The knowledge of prevention and control of echinococcosis was investigated in villages to evaluate the change in population's TKAR. The contents of the investigation include five core knowledge points: What kind of disease is echinococcosis? (WK-ID), how do humans, cattle, and sheep get infected with echinococcosis? (HH-WE), how do dogs get infected with echinococcus? (HD-WE), how to prevent dogs from transmitting hydatid disease to humans? (HT-TP), how to prevent echinococcosis? (HT-PE), those who can answer three or more of the five questions correctly are qualified.

#### 2.3.3. Situation of People Raising Dogs

Conduct an investigation on dog rearing methods(CA-RM), STDA, DRDD, whether SDFD has been fed in the last 3 months, and whether diseased organs have been fed directly to animals in the village unit, then the results were used to evaluate the change of dog-keeping behavior among the population.

#### 2.3.4. Survey of Crowd Behavior Habits

Conduct a survey on the living habits of the population within the village unit, such as contact with dogs, handwashing practices, consumption of raw water, and burning cow dung. Then, evaluate the changes in the living habits of the population with the survey results.

#### 2.3.5. Population Attitude Survey

To investigate whether people are willing to keep dogs in isolation, whether they are willing to administer deworming drugs, and whether they will feed diseased organs directly to dogs in the village unit, the survey results were then used to evaluate the attitude of the population towards the prevention and control of echinococcosis.

#### 2.3.6. Statistical

Confirmatory factor analysis (CFA) was used to test the structural validity of the EC-DD Scale. This study was divided into two groups (Set 1, TIG, 3 villages; Set 2, TCG, 1 village).

The grouping is as follows: TKAR (WK-ID: 1 = “*infectious diseases transmissible from person to person (ID-TP)*,” 2 = “*It is an infectious disease transmitted from cattle to humans (II-TH)*,” 3 = “*diseases caused by malnutrition*” (DCBM), 4 = “*It is caused by worm eggs in dog feces entering the human body (II-HB)*,” and 5 = “*a disease caused by insect bites in the grasslands (AD-TG)*”; HH-WE: 1 = “*contact with a person or animal with hydatid disease (CW-HD)*,” 2 = “*ingestion of worm eggs from feces of sick dogs (IO-SD)*,” 3 = “*eating raw diseased organs of animals (ER-OA)*,” and 4 = “*eating raw meat*”; HD-WE: 1 = “*eating the feces of a patient with hydatid disease*,” 2 = “*eating raw diseased organs of domestic animals (ER-DA)*,” and 3 = “*exposure to dogs with hydatid disease (ET-WH)*”; HT-TP: 1 = “*regular deworming of the dog (RD-TD)*,” 2 = “*feeding the dogs raw offal (FT-RO)*,” 3 = “*spraying the dog with insecticide (ST-WI)*”, and 4 = “*prevention of dog bites (PODB)*”; HT-PE: 1 = “*Do not eat raw beef and mutton (DN-AM)*,” 2 = “*Wash your hands before eating, don't play with dogs (WY-WD)*,” and 3 = “*Use mosquito nets to prevent bites (UM-PB)*”.).

People with dogs are as follows: (Do you or have you ever had dogs in your family (DY-YF): 1 = “*have*”, 2 = “*have not*”, 3 = “*had before*,” and 4 = “*never*”; the way of family dog rearing(TW-DR): 1 = “*stocking*”, 2 = “*tethering*”, and 3=“*sometimes stocking or tethering*”; the way of family dog tethering (TW-DT): 1 = “*alone*,” 2 = “*together with cattle and sheep*,” and 3 = “*together with pigs*”; how to deal with the dog poop (HT-DP): 1 = “*garbage disposal*,” 2 = “*no processing*,” and 3 = “*buried or burned*”; has your family dog been treated with insect repellent in the past 3 months(HY-TM): 1 = “*yes*,” 2 = “*no*,” and 3=“*I don't remember*”; has your family dog ever been fed raw animal organs (HY-AO): 1 = “*yes*,” 2 = “*no*,” and 3 = “*I do not remember*”).

Crowd life behavior habits are as follows: contact with dog: 1 = “*no contact*”, 2 = “*touch it occasionally*”, 3 = “*embrace often or stroke*”; Do you wash your hands before preparing meals or eating (DY-OE): 1 = “*not washing*”, 2 = “*wash occasionally*,” and 3 = “*wash frequently*”; did your family use cow dung to burn (DY-TB): 1 = “*use*,”, 2 = “*do not use*”; have you drunk wild water (HY-WW): 1 = “*yes*,” 2 = “*no*,” and 3 = “*I don't remember*”).

Attitude of the crowd is as follows: (Would you like your dog to be kept separately (WY-KS): 1 = “*willing*” and 2 = “*unwilling*”; Would you like to give the dog insect repellent(WY-IR): 1 = “*willing*” and 2 = “*unwilling*”; Would you directly feed the dog with diseased organs from livestock(WY-FL): 1 = “*yes*” and 2 = “*no*”.)

All data were inputted using double entry in the Excel database with error correction by double-entry comparison. The rate of infection in dogs with 95% confidence intervals (CI) was calculated by SPSS 17.0 (IBM, New York, United States). The data are expressed as frequency and percentage, and the comparison of rates between groups was tested using the chi-square test, which was performed. The significance level was set as *p* < 0.05.

## 3. Results

### 3.1. Core Measures

In TIG, RMDD was tied alone and did not run away, and dogs were not kept in captivity with cattle, sheep, pigs, and other domestic animals, SDFD within 3 days after deworming. Among them, 277 dogs were selected for registration management and deworming observation in Jiefang Village, deworming once a month, a total of 3324 deworming, and 8940 dog feces were buried deep. In Nishi Village, 407 dogs were selected for registration management and deworming observation, deworming once a quarter, a total of 1628 times, and 4589 dog feces were buried deep. In Jidi Village, 385 dogs were selected for registration management and deworming observation, deworming once every 6 months, a total of 770 deworming times, and 2130 dog feces were buried deep. Village is a unit that carries out health publicity through on-site publicity, distribution of echinococcosis brochures, publicity items, village WeChat group forward propaganda short video, and other ways. In TIG, a total of 3500 copies of echinococcosis prevention and control knowledge pamphlets, 1200 boxes of paper, 4000 paper cups, 800 cloth bags, and 500 aprons were distributed, and the beneficiary population covers about 3800.

### 3.2. Canine Infection Investigation

**Figure 2:**
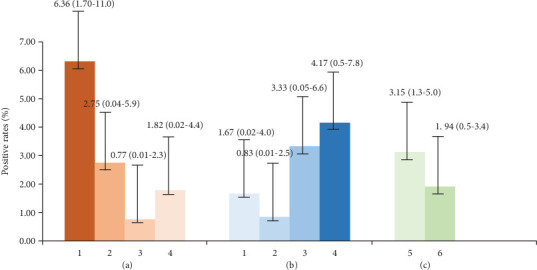
Positive rates of canine infection. (a) Baseline value: TIG ((1) Jiefang Village, (2) Nishi Village, and (3) Jidi Village) and TCG ((4) Hongpo Village); (b) Evaluation value: TIG ((1) Jiefang Village, (2) Nishi Village, and (3) Jidi Village) and TCG ((4) Hongpo Village); (c) TIG ((5) baseline survey and (6) assessment survey).

The positive rates of fecal antigen in TIG and TCG were 1.94% and 4.17%, respectively. Compared with the baseline value of 3.15% PREA in the intervention group decreased by 38.41%. In contrast, the PREA of TCG increased by 56.35% compared with the baseline value of 1.82%. Among the villages assessed, the PREA of Jiefang Village was 1.67%, which decreased by 73.74% compared with the baseline of 6.36%. The PREA of the Nishi Village assessment survey was 0.83%, which was 69.82% lower than the baseline of 2.75%. However, the PREA of the Jidi village assessment survey was 3.33%, which was 77.00% higher than the baseline of 0.77% (Figure 2).

### 3.3. Survey of TKAR of Population

TIG and TCG adopted the field questionnaire survey method, in which those who could correctly answer 3 relevant questions were qualified. The estimated average TKAR of the population was 81.46% (391/480), which was increased by 30.57% compared with the baseline of 56.56% (250/442). Compared with the baseline value, the TKAR of the population in Jidi, Nishi, and Jiefang villages increased by 15.79%, 25.30%, and 23.08%, respectively. The TKAR of TCG in Hongpo Village increased by 5.55%. About the five core knowledge, the TKAR of WK-ID (82.08%, 394/480) increased by 33.30% compared with the baseline value (54.75%, 242/442). HH-WE (78.33%, 376/480) increased by 66.79% compared with the baseline value (26.02%, 115/442). The estimated knowledge of HD-WE increased by 33.30% (70.42%, 338/480) compared with the baseline (49.10%, 217/442). How does the knowledge of HT-TP increase by 33.30% (82.08%,394/480) compared to the baseline (62.44%, 276/442)? The estimated knowledge of HT-PE (80.21%, 385/480) increased by 33.30% compared with the baseline value (74.21%, 328/442) (Table 1).

### 3.4. Survey on People Raising Dogs

**Figure 3:**
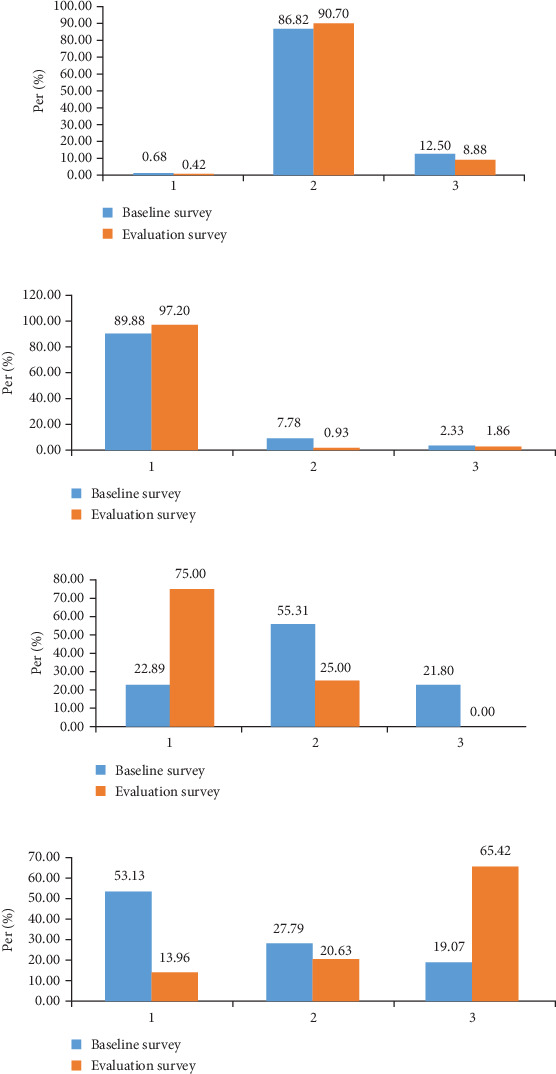
Survey of dog ownership behavior among the population. (a) How dogs are kept ((1) free range, (2) leash, and (3) sometimes free range and sometimes leash); (b) how dogs are kept ((1) alone, (2) with cattle and sheep, and (3) with pigs); (c) domestic dog deworming situation ((1) yes, (2) no, and (3) do not remember whether deworming); (d) dog manure disposal ((1) garbage disposal, (2) no disposal, and (3) deep burial or incineration treatment).

In the baseline survey, 66.97% (296/442) of households had a dog, 11.54% (51/442) did not have a dog, and 5.43% (24/442) had never had a dog. The methods of dog rearing, dog tying, dog manure treatment, whether the diseased animal organs were fed to the dog, and whether the deworming drugs were fed within the past 3 months were investigated. The evaluation values for free-range, leashed, and sometimes free-range or leashed accounted for 0.42% (2/473), 90.70% (429/473), and 8.88% (42/473), respectively. The leashed dog assessment value of 90.70% (429/473) increased by 4.28% compared to baseline value of 86.82% (257/298) (Figure 3(a)). In the case of dog tying, the proportions of dogs being tied alone, kept with cattle and sheep, and kept with pigs were 97.20% (417/429), 0.93% (4/429), and 1.86% (8/429), respectively. The assessed value of dogs leashed alone was 97.20% (417/429), up 7.34% from the baseline value of 89.88% (231/257) (Figure 3(b)). Among the dogs that had been fed deworming drugs in the past 3 months, the proportions of those that had, had not, and could not remember whether they had been deworming were 75.00% (360/480), 25.00% (120/480), and 0.00% (0/480), respectively. The assessed value of dog deworming increased by 69.48% compared to the baseline value of 22.89% (84/367) (Figure 3(c)). In dog waste treatment, the proportion of evaluation values of garbage disposal, deep burial or incineration, and no treatment were 13.69% (67/480), 65.42% (314/480), and 20% (99/480), respectively. The assessed value of deep burial or incineration of dog waste was 65.42% (314/480) compared to the baseline value of 19.07% (70/367), an increase of 70.74%(see Figure 3(d)). Regarding whether the dogs were fed diseased organs of the animals, the proportion of assessment values that were, were not, and could not remember whether they had been fed were 2.92% (14/480), 97.08% (466/480), and 0% (0/480), respectively. The proportion of dogs that had not been fed diseased organs increased by 42.75% compared to the baseline value of 55.58% (205/367).

### 3.5. Survey of Life and Behavior Habits of the Population

There was a 41.31% increase from baseline in people not touching dogs and a 20.07% decrease in those occasionally touching dogs. Compared with the baseline, the assessment that people regularly washed their hands increased by 19.70%, the estimated value of drinking raw water decreased by 30.42%, and the value of not drinking raw water increased by 40.21%. The estimated value of household fires without cow dung increased by 7.84% compared to the baseline value (Table 2).

### 3.6. Population Attitude Assessment Survey

In each village, the people were asked whether they were willing to keep dogs alone, whether they were willing to deworm dogs, and whether they would feed the dogs with diseased organs. On the question of whether they would like to keep a dog alone, 98.10% (464/473) and 1.90% (9/473) of the assessed values agreed and did not agree, respectively. About whether they are willing to dewormer dogs, the proportion of willing and unwilling evaluation values was 98.94% (468/473) and 1.06% (5/473), respectively. As to whether they would feed diseased organs directly to dogs, the assessment values of yes and no were 0.63% (3/473) and 99.37% (470/473), respectively.

## 4. Discussion

In 1978, the Surgery Department of Yunnan Province reported more than 10 cases of echinococcosis. Zhang Bingxiang et al. reported a total of 24 surgically confirmed cases of Echinococcosis in Yunnan Province from 1981 to 2022, primarily occurring in Diqing Tibetan Autonomous Prefecture and Dali Bai Autonomous Prefecture [25–28]. The survey results conducted by Li Benfu et al. indicated that the prevalence of echinococcosis in Yunnan Province was moderate to low, mainly distributed across 24 counties (cities and districts) in nine prefectures (cities and districts) located northwest of the 25° north latitude, including Diqing and Dali. The detection rate of echinococcosis among the population was 0.06%. The investigation confirmed the existence of a natural epidemic source in Yunnan, with the epidemic circulating between dogs and animals, posing a potential risk of human infection. The fecal antigen-positive rate of dogs in Diqing Shangri-La was 10.65%, significantly higher than the national average [19]. Dogs are the most common definitive hosts of echinococcosis, and they have been identified as the primary source of human CE infection [29]. A study by Campos-Bueno, Lopez-Abente, and Andres-Cercadillo [30] showed that owning dogs was associated with an increased risk of human echinococcosis.

Dog ownership behavior, number of dogs owned, and stray dogs are key risk factors for Echinococcosis [31–38]. However, the infection of domestic dogs is related to human life habits, such as allowing dogs to roam freely and feeding diseased organs of privately slaughtered animals directly to dogs, which increase the chances of Echinococcus infection in dogs [39, 40]. The infection of domestic dogs is related to human life and behavior. For example, allowing dogs to roam freely and directly feeding them diseased organs from privately slaughtered animals elevates the likelihood of Echinococcus infection in dogs, thereby establishing a cycle of transmission between dogs and other domesticated animals like cattle, sheep, and pigs. After direct or indirect contact with dogs, cattle, sheep, pigs, and other animals, humans may accidentally ingest the eggs and become infected. This increases the risk of echinococcosis in the population [37, 41, 42]. The eggs develop into hydatid cells in human or domesticated animals, usually causing localized infections, mainly parasitizing in the liver, the early symptoms of infection are not obvious, but as the hydatid cyst infection progresses, it can become very serious and even fatal, affecting the health of the host [43, 44]. A study conducted in Shiqu County reported that risk factors for echinococcosis in a population include age, sex, dog ownership, and drinking water sources [45, 46]. Stray dogs are significant vectors for the transmission of echinococcosis, and managing stray dogs effectively or keeping them away from residential areas may reduce the incidence of echinococcosis [47].

Therefore, the prevention and control of infectious dogs is the key to the prevention and control of echinococcosis, and deworming is a direct measure to reduce the infection rate of echinococcosis in dogs. For areas with a high prevalence of echinococcosis, canine deworming and monthly deworming are important means. In areas with a low prevalence of echinococcosis, the dog infection rate is low or even zero in most regions except for local positive dogs. Therefore, canine deworming and monthly deworming measures may not be the best solution. The reasons are as follows: First, it is a waste of time and money, coupled with the lack of grassroots health workers, which does not guarantee the quality of deworming; second, the investment is too limited to cover all dogs' deworming needs; third, rural residents are busy with production activities, making it difficult to implement monthly deworming for dogs; fourth, the disposal of dog feces after deworming is also a challenge. If feces are not properly buried or burned, *Echinococcus* eggs may survive, thereby expanding the scope of egg contamination and increasing the risk of infection in the population.

The results of this study showed that in the village where the positive rate of dog *Echinococcus* fecal antigen was more than 5%, deworming was taken once a month and 12 times a year, and the positive rate was less than 5%. And the positive rate of fecal antigen of Echinococcus hydatid in dogs decreased significantly in more than 1% of villages, which used dog deworming once a quarter and 4 times a year. The positive rate in villages with less than 1% was dewormed once every 6 months, and the positive rate of fecal antigen of *Echinococcus* Canis increased compared with the control group without deworming. Therefore, in areas with low prevalence of echinococcosis, the positive rate of dogs can be effectively controlled by deworming dogs at least once a quarter and more than 4 times a year. Deworming intervention combined with population health intervention and other comprehensive prevention and control measures can improve people's awareness of hydatid disease and change people's behavior habits, such as strict management of dogs, not hiring dogs, washing hands frequently, and not drinking raw water, so as to reduce the risk of hydatid infection.

As previously mentioned, humans become “victims” because of consuming proglottid or eggs in contaminated food or water. The results of this study showed that some factors related to the living habits, such as “not washing hands before meals,” “drinking nonboiled water,” and “eating raw vegetables,” may contribute to increased risk, results that were consistent with those of previous studies [48, 49].

## 5. Conclusion

Overall, echinococcosis is one of the key diseases for prevention and control in Yunnan. Based on the discovery of cases, an epidemiological investigation was conducted. Subsequently, according to the investigation results, ultrasonic screening of human echinococcosis, infection investigation of domestic dogs, deworming, and registration management were implemented in the villages identified as the epidemic foci of local infections. Then, health interventions were combined to improve the living environment and behavior, enhancing self-protection awareness and treatment knowledge. For epidemic foci of imported infection cases, the main focus is to investigate the source of infection, determining, whether the case was infected directly from an endemic area or after contact with dogs, cattle, sheep, pigs, or fur from an endemic area. To investigate imported domestic animals, when positive dogs or other infected animals are found, they are regulated and treated. Further health intervention measures are then taken to effectively control the transmission of echinococcosis.

## Figures and Tables

**Table 1:** Baseline and assessment survey of core knowledge of hydatid disease control.

**Core knowledge**	**Pass rate % (baseline)**	**Pass rate % (evaluation)**	**χ**2	**p**
WK-ID	54.75 (242/442)	82.08 (394/480)	78.906	≤ 0.000
HH-WE	26.02 (115/442)	78.33 (376/480)	252.982	≤ 0.000
HD-WE	49.10 (217/442)	70.42 (338/480)	43.659	≤ 0.000
HT-TP	62.44 (276/442)	82.08 (394/480)	113.562	≤ 0.000
HT-PE	74.21 (328/442)	80.21 (385/480)	4.267	> 0.001

**Table 2:** Baseline and assessment survey of life and behavior habits of the population.

**Survey content**		**Percentage %(baseline)**	**Percentage % (evaluation)**	**χ**2	**p**
Contact with dogs	1 = no contact	22.62 (100/442)	38.54 (185/480)	27.299	≤ 0.000
	2 = occasionally touch	73.76 (326/442)	58.96 (283/480)	22.470	≤ 0.000
	3 = often hug/touch	2.26 (10/442)	2.50 (12/480)	0.056	> 0.000

Do you wash your hands before eating?	1 = no hand washing	4.75 (21/442)	3.96 (19/480)	0.348	> 0.000
	2 = washing hands occasionally	58.60 (259/442)	50.42 (242/480)	6.207	> 0.000
	3 = frequent hand washing	36.65 (162/442)	45.63 (219/480)	7.642	> 0.000

Do you have cow dung fires in your home?	1 = yes	8.60 (38/442)	0.83 (4/480)	31.900	≤ 0.000
	2 = no	91.40 (404/442)	99.17 (476/480)		

Have you ever drunk raw water?	1 = yes	61.99 (274/442)	43.13 (207/480)	32.822	≤ 0.000
	2 = no	30.77 (136/442)	51.46 (247/480)	40.599	≤ 0.000
	3 = do not remember	7.24 (32/442)	6.67 (32/480)	0.117	> 0.000

## Data Availability

All data can be obtained from the corresponding authors.

## References

[1] Craig P. S., Deshan L., Zhaoxun D. (1991). Hydatid disease in China. *Parasitology Today*.

[2] Romig T., Deplazes P., Jenkins D. (2017). Ecology and life cycle patterns of Echinococcus species. *Advances in Parasitology*.

[3] Cadavid Restrepo A. M., Yang Y. R., McManus D. P. (2016). The landscape epidemiology of echinococcoses. *Infectious Diseases of Poverty*.

[4] Budke C. M., Deplazes P., Torgerson P. R. (2006). Global socioeconomic impact of cystic echinococcosis. *Emerging Infectious Diseases*.

[5] Zhang M. Y., Wu W. P. (2017). Advances in study the burden of echinococcosis in China and elsewhere around the world. *Journal of Pathogen Biology*.

[6] Xiao N., Yao J.-W., Ding W., Giraudoux P., Craig P. S., Ito A. (2013). Priorities for research and control of cestode zoonoses in Asia. *Infectious Diseases of Poverty*.

[7] Kirigia J. M., Mburugu G. N. (2017). The monetary value of human lives lost due to neglected tropical diseases in Africa. *Infectious Diseases of Poverty*.

[8] McManus D. P., Zhang W., Li J., Bartley P. B. (2003). Echinococcosis. *Lancet*.

[9] Moro P., Schantz P. M. (2009). Echinococcosis: a review. *International Journal of Infectious Diseases*.

[10] World Health Organization (2010). *Working to overcome the global impact of neglected tropical diseases, first WHO report on neglected tropical diseases*.

[11] Torgerson P. R., Devleesschauwer B., Praet N. (2015). World Health Organization estimates of the global and regional disease burden of 11 foodborne parasitic diseases, 2010: a data synthesis. *PLoS Medicine*.

[12] Ito A., Urbani C., Jiamin Q. (2003). Control of echinococcosis and cysticercosis: a public health challenge to international cooperation in China. *Acta Tropica*.

[13] Xu J., Xu J.-F., Li S.-Z. (2015). Integrated control programmes for schistosomiasis and other helminth infections in P.R. China. *Acta Tropica*.

[14] Zhang W., Zhang Z., Wu W. (2015). Epidemiology and control of echinococcosis in Central Asia, with particular reference to the People’s Republic of China. *Acta Tropica*.

[15] World Health Organization Report of the WHO informal working group on cystic and alveolar echinococcosis surveillance, prevention and control, with the participation of the Food and Agriculture Organization of the United Nations and the World Organisation for Animal Health. http://apps.who.int/iris/bitstream/10665/44785/1/9789241502924_eng.pdf.

[16] Grabellus F., Worm K., Schmid K. W. (2007). Induction of the matrix metalloproteinase-2 activation system in arteries by tensile stress. Involvement of the p38 MAP-kinase pathway. *Pathology, Research and Practice*.

[17] MOH (Ministry of Health, China) (2007). *Report on the national survey of current status of major human parasitic diseases in China*.

[18] Yang J., Liu T. X. (2008). Progress in epidemiological research on hydatidosis. *Ningxia Medical Journal*.

[19] Wu W. P., Wang H., Wang Q. (2018). A nationwide sampling survey on echinococcosis in China during 2012-2016. *Chinese Journal of Parasitology and Parasitic Diseases*.

[20] Benfu L. I., Shuai S. H. I., Wenshen H. E. (2023). Epidemiological analysis of echinococcosis in Diqing Tibetan Autonomous Prefecture from 2016 to 2020. *Journal of Pathogen Biology*.

[21] Guralp N. (1981). *Helmintology*.

[22] Kaufmann J. (1996). Parasitic infections of domestic animals. *A diagnostic manual*.

[23] Rinaldi F., Brunetti E., Neumayr A., Maestri M., Goblirsch S., Tamarozzi F. (2014). Cystic echinococcosis of the liver: a primer for hepatologists. *World Journal of Hepatology*.

[24] Zi J. R., Xiao D., Peng J. (2024). Epidemiological survey of cystic echinococcosis in Southwest China: from the Qinghai-Tibet Plateau to the area of Yunnan. *World Journal of Hepatology*.

[25] Zhang B. X., Zhang L. L., Yang H. M. (1997). Investiagtion of hydatid diseases in Yunnan province. *Chinese Journal of Zoonoses*.

[26] Pang Y. K. (2004). Analysis of hydatid disease data during 1981-2001 in Yunnan province. *Chinese Journal of Parasitic Disease Control*.

[27] Li B. F., Wu F. W., Yan X. L. (2019). Epidemiological analysis of echinococcosis in Yunnan Province from 2012 to 2017. *Chinese Journal of Parasitology and Parasitic Diseases*.

[28] Li B. F., He W. S., Zi J. R. (2020). Analysis of the prevalence of and control measures for echinococcosis in Shangri La, Yunnan Province. *Journal of Pathogen Biology*.

[29] Craig P. S., Rogan M. T., Campos-Ponce M. (2003). Echinococcosis: disease, detection and transmission. *Parasitology*.

[30] Campos-Bueno A., Lopez-Abente G., Andres-Cercadillo A. M. (2000). Risk factors for Echinococcus granulosus infection: a case-control study. *The American Journal of Tropical Medicine and Hygiene*.

[31] Craig P. S., Giraudoux P., Shi D. (2000). An epidemiological and ecological study of human alveolar echinococcosis transmission in South Gansu, China. *Acta tropica*.

[32] Liwk S. D. Z., Bao G. S. (2003). Prevalence of alveolar echinococcosis in Gansu Province and human behavior risk factors. *China Public Health*.

[33] Shi D. Z., Zhao Y. M., Guo Z. H. (2004). Prevalence and risk factor analysis of alveolar echinococcosis in Dingxi prefecture of Gansu Province. *Chinese Journal of Zoonoses*.

[34] Possenti A., Manzano-Román R., Sanchez-Ovejero C. (2016). Potential risk factors associated with human cystic echinococcosis: systematic review and meta-analysis. *PLoS Neglected Tropical Diseases*.

[35] Wang Q., Vuitton D. A., Qiu J. M. (2004). Fenced pasture: a possible risk factor for human alveolar echinococcosis in Tibetan pastoralist communities of Sichuan, China. *Acta Tropica*.

[36] Zhao Y. M., Jing T., Ma S. M. (2010). A study of the prevalence of human echinococcosis in Maqu and Luqu counties of Gannan Tibetan Autonomous Prefecture, China. *Journal of Pathogen Biology*.

[37] Wang Q., Qiu J., Yang W. (2006). Socioeconomic and behavior risk factors of human alveolar echinococcosis in Tibetan communities in Sichuan, People’s Republic of China. *The American Journal of Tropical Medicine and Hygiene*.

[38] Wang Q., Xiao Y. F., Vuitton D. A. (2007). Impact of overgrazing on the transmission of Echinococcus multilocularis in Tibetan pastoral communities of Sichuan Province, China. *Chinese Medical Journal*.

[39] Wei H. E., Wenjie Y. U., Yan H. U. (2023). Effectiveness of comprehensive echinococcosis control measures with emphasis on management of infectious source in Sichuan Province from 2010 to 2022. *Chinese Journal of Schistosomiasis Control*.

[40] Fu Y. J., Wang S. X., Lin Y. Q., Li X. Y. (2015). Echinococcosis in cattle and sheep in QingHai province, China. *Animal Husbandry & Veterinary Science*.

[41] Schantz P. M., Wang H., Qiu J. (2003). Echinococcosis on the Tibetan Plateau: prevalence and risk factors for cystic and alveolar echinococcosis in Tibetan populations in Qinghai Province, China. *Parasitology*.

[42] Kassai T. (1999). *Veterinary helminthology*.

[43] Senlik B. (2008). Influence of host breed, sex and age on the prevalence and intensity of Cysticercus tenuicollis in sheep. *Journal of Animal and Veterinary Advances*.

[44] Schineider T. (2006). *Veterinary parasitology*.

[45] Omar M. A., Elmajdoub L. O., Al-Aboody M. S., Elsify A. M., Elkhtam A. O., Hussien A. A. (2016). Molecular characterization of Cysticercus tenuicollis of slaughtered livestock in Upper Egypt governorates. *Asian Pacific Journal of Tropical Biomedicine*.

[46] Tiaoying L., Jiamin Q., Wen Y. (2005). Echinococcosis in Tibetan populations, western Sichuan Province, China. *Emerging Infectious Diseases*.

[47] Li T., Chen X., Zhen R. (2010). Widespread co-endemicity of human cystic and alveolar echinococcosis on the eastern Tibetan Plateau, Northwest Sichuan/Southeast Qinghai, China. *Acta Tropica*.

[48] Yang Y. R., Sun T., Li Z. (2006). Community surveys and risk factor analysis of human alveolar and cystic echinococcosis in Ningxia Hui Autonomous Region, China. *Bulletin of the World Health Organization*.

[49] Harandi M. F., Moazezi S. S., Saba M. (2011). Sonographical and serological survey of human cystic echinococcosis and analysis of risk factors associated with seroconversion in rural communities of Kerman, Iran. *Zoonoses Public Health*.

